# Intranasal Delivery of Chitosan Nanoparticles for Migraine Therapy

**DOI:** 10.3797/scipharm.1208-18

**Published:** 2013-04-14

**Authors:** Neha Gulati, Upendra Nagaich, Shubhini A. Saraf

**Affiliations:** 1 Department of Pharmaceutics, School of Pharmacy, Bharat Institute of Technology, Meerut, UP, India.; 2 School of Biosciences and Biotechnology, Babasaheb Bhimrao Ambedkar University, Lucknow, India.

**Keywords:** Sumatriptan succinate, Taguchi design, Goat nasal mucosa, Tween 80, Sodium tripolyphosphate, Ionotropic Gelation

## Abstract

**Objective:**

The objective of the research was to formulate and evaluate sumatriptan succinate-loaded chitosan nanoparticles for migraine therapy in order to improve its therapeutic effect and reduce dosing frequency.

**Material and Methods:**

The Taguchi method design of experiments (L9 orthogonal array) was applied to obtain the optimized formulation. The sumatriptan succinate-loaded chitosan nanoparticles (CNPs) were prepared by ionic gelation of chitosan with tripolyphosphate anions (TPP) and Tween 80 as surfactant.

**Results:**

The CNPs had a mean size of 306.8 ± 3.9 nm, a zeta potential of +28.79 mV, and entrapment efficiency of 75.4 ± 1.1%. The *in vitro* drug release of chitosan nanoparticles was evaluated in phosphate buffer saline pH 5.5 using goat nasal mucosa and found to be 76.7 ± 1.3% within 28 hours.

**Discussion:**

The release of the drug from the nanoparticles was anomalous, showing non-Fickian diffusion indicating that drug release is controlled by more than one process i.e. the superposition of both phenomena, a diffusion-controlled as well as a swelling-controlled release. This is clearly due to the characteristics of chitosan which easily dissolves at low pH, thus a nasal pH range of 5.5 ± 0.5 supports it very well. The mechanism of pH-sensitive swelling involves protonation of the amine groups of chitosan at low pH. This protonation leads to chain repulsion, diffusion of protons and counter ions together with water inside the gel, and the dissociation of secondary interactions.

**Conclusion:**

The results suggest that sumatriptan succinate-loaded chitosan nanoparticles are the most suitable mode of drug delivery for promising therapeutic action.

## Introduction

Numerous drug delivery systems have been discovered as therapies for brain disorders which may improve efficacy and reduce the toxic effects of the active compounds. The blood-brain barrier (BBB) is the most vital element present in the central nervous system which selectively permits the access of preferred molecules from the blood to the brain [1]. Nanoparticles made of erodible polymers have turned out to be the preferred drug delivery vehicles because of their biodegradable nature and ease in removal from the system after drug delivery [2]. Nanoparticles (NPs) can be defined as submicron colloidal drug carrier systems which are composed of natural or artificial polymers ranging in size between 10 and 100 nm[3]. Nanoparticles have several advantages over other novel delivery systems (microparticles, microemulsions, nanoemulsions etc.) such as site-specific targeting, prevention of dose dumping via sustained and controlled release, and high surface-to-volume ratio. These attributes indirectly help in reducing dose and frequency of administration which improves patient compliance [4]. Chitosan is a biodegradable, non-toxic, non-immunogenic polymer, abundantly found in nature and also approved as GRAS (Generally Recognized as Safe by the United States FDA) [5]. Chitosan is a natural polysaccharide obtained from the partial deacetylation of chitin from crustacean cells. Chitosan nanoparticles are prepared by a mild preparation technique known as ionotropic gelation. The mechanism of nanoparticle formation is through the ionic interaction between oppositely charged species like the positive groups of chitosan (amino groups) and negative groups of sodium tripolyphosphate (hydroxyl and phosphoric ions) at room temperature [6]. The preeminent attribute of the method used is drug entrapment in aqueous and physiological conditions. This does not involve the use of organic solvents/chemicals like glutaraldehyde, chloroform etc. or any chemical modifications. The positive groups of chitosan enhance its mucoadhesive nature, since it can bind to negatively charged mucosal secretions of the nasal cavity; thus aiding in intranasal drug delivery for the therapy of brain diseases like migraines, Alzheimer’s, epilepsy, tumors, and so on [7].

A migraine is a common headache disorder which significantly affects about 15% of females and 6% of males. It is a neurobiological syndrome which is mainly characterized by a unilateral throbbing headache [8]. Other major symptoms include nausea, vomiting, and sensitivity to light. During the migraine attack, blood vessels of the brain get dilated due to a decrease in the level of the vasoconstrictor known as 5-hydroxytryptamine (5-HT) which causes intense headaches [9]. Sumatriptan succinate is a triptan derivative which is a serotonin agonist used as the main drug in migraine therapy. The major drawback of this drug is its low oral absolute bioavailability (only 15%) which may be attributed to its extensive first pass metabolism [10]. Therefore, to deliver the required dose of drug to the brain, an alternative route is proposed which is the nasal route. This route is practical, non-invasive, and also circumvents the problem of dose wastage which may occur due to emesis during a migraine attack. Drug delivery via intranasal route helps in bypassing the blood-brain barrier (BBB), whereby direct brain targeting can be achieved with absorption through the olfactory mucosa [11]. In the present work, chitosan nanoparticles were formulated as the delivery vehicle for the antimigraine drug, sumatriptan succinate, and were proposed to be given through the nasal route.

## Materials and Methods

### Materials

Sumatriptan succinate was received as a gift sample from Sun Pharmaceutical Ind. Ltd., Dadra and Nagar Haveli, India. Chitosan (85% deacetylated) was purchased from Sigma Aldrich Pvt. Ltd., Mumbai, India. Sodium tripolyphosphate and Tween 80 were purchased from Loba Chemie Pvt. Ltd. (Mumbai, India) and S.D Fine-Chem Pvt. Ltd. (Mumbai, India), respectively. All other reagents were of analytical grade and used as purchased.

### Statistical Experimental Design for Formulation Optimization

The Taguchi method design of experiments is a statistical tool which mainly relies upon the systematic approach of conducting a minimum number of experiments with the use of a mathematical instrument known as orthogonal arrays (OA) [12]. The method is primarily utilized to envisage the contribution of each variable and its level to attain an optimum combination. The method also gives a full description of all the factors that affect the performance parameters. Based on the number of factors and their levels, an L_9_ (3^4^) orthogonal array was employed [13]. The four factors (formulation variables) i.e. polymer concentration (%w/v), cross-linking agent concentration (%w/v), surfactant concentration (%v/v), and stirring speed (rpm) were selected. All the factors were assigned three levels i.e. low, medium, and high as shown in Table 1. The L9 orthogonal array is explained in Table 2, which describes the number of formulations to be developed for optimization. Apart from designing a formulation table, optimizations were done on the basis of two main approaches: the “smaller-the-better” and “larger-the-better”, and the signal-to-noise ratio (S/N ratio). The S/N ratio takes into account the mean and variation between results [14]. The “smaller-the-better” is usually the chosen S/N ratio for all undesirable characteristics like “defects” etc. for which the ideal value is zero. Examples are the size of nanoparticles and polydispersity index, while the “larger-the-better” is taken when the desired ideal value is larger. Examples are drug entrapment efficiency and drug-loading etc. [15].

### Preparation of Sumatriptan Succinate-loaded Chitosan Nanoparticles

Chitosan nanoparticles containing the drug sumatriptan succinate were prepared by the ionotropic gelation technique [16]. Accurately weighed chitosan was dissolved in 1% v/v acetic acid solution, to which Tween 80 and the drug were added. Sodium tripolyphosphate (TPP) was dissolved in distilled water. To the chitosan-drug solution, TPP solution was added dropwise through a no. 4 syringe needle and continuously stirred using a mechanical stirrer (Remi Motors- RO-123, RPM 4000) at room temperature for 30 min, which led to the formation of nanoparticles. Subsequently, the pH was adjusted to 5.5 with the help of a required amount of 1N HCl or NaOH and then centrifuged at 12000 rpm using a refrigerated centrifuge (SIGMA 3-18K, Sartorius)[17].

### Characterization of Chitosan Nanoparticles

#### Shape and Surface morphology

The shape and surface morphology of the chitosan naoparticles was visualized by scanning electron microscopy (LEO-430 Cambridge and U.K). The samples were prepared by lightly sprinkling nanoparticles on double-sided adhesive tape on an aluminum stub. The stubs were then coated with gold to a thickness of 200 to 500 Å under an argon atmosphere using a gold sputter module in a high vacuum evaporator. The samples were then randomly scanned and photomicrographs were taken at different magnifications with SEM [17].

#### Particle Size and Zeta Potential Measurement

Particle size was measured with the help of photon correlation microscopy. For the determination of particle size, samples were prepared by tenfold dilution of 1ml of the nanoparticulate suspension with distilled water. The analysis was carried out in triplicate. The average particle size and polydispersity index were measured by photon correlation spectroscopy. Zeta potential was determined by the electrophoretic mobility of nanoparticles in U-type tube at 25°C, using a zetasizer (3000HS Malvern Instruments, UK) [18].

#### Drug Entrapment Efficiency

The entrapment efficiency of the formulation was determined upon the centrifugation of a fixed quantity of the aqueous nanoparticulate suspension (about 10ml) at 12000 rpm for 30 minutes at 20°C (SIGMA 3-18K, Sartorious). The absorbance of the unencapsulated drug was evaluated in the supernatant using a UV-VIS spectrophotometer (UV-1700 PharmaSpec, Shimadzu) at 283 nm using a calibration curve with plain chitosan nanoparticles (CNPs) as the blank which had also been prepared and treated similarly to the drug-loaded nanoparticles [19]. The analysis was carried out in triplicate and the mean was taken. The drug entrapment of the nanoparticles was calculated by the following equation.

$$\%\;\text{Drug Entrapment efficiency}=\frac{\text{Amount of drug in supernatant}}{\text{Initial amount of drug added}}\times 100$$

#### In vitro Drug Release Studies

*The in vitro* drug release study was conducted using a Keshary-Chien (K-C) cell with an effective diffusion area of 2.0 cm^2^ and a cell volume of 25 ml. The diffusion cells were thermoregulated with a water jacket at 37±2°C. Excised goat nasal mucosa was used for the evaluation of the formulation’s permeation which was obtained from a local slaughter house within 15 minutes of the goats’ sacrifice. After skin removal, the nose was stored on an ice-cold phosphate buffer (pH 5.5) and nasal mucosa was carefully removed using forceps and surgical scissors. The mucosal tissues were immediately immersed in Ringer’s solution. The freshly excised nasal mucosa was mounted on the diffusion cell and 10 ml of the aqueous drug-loaded nanoparticulate suspension (containing drug equivalent to 10 mg) was kept on it. The receptor chamber was filled with fresh saline (phosphate buffer pH 5.5). One ml of sample aliquots were withdrawn at predetermined time intervals and subsequently replenished with an equal amount of phosphate buffer. The samples were filtered and diluted appropriately. The samples were analyzed spectrophotometrically at 283nm [20, 21].

#### Release Kinetics Studies

The release kinetics of the drug was studied by plotting the results of the *in vitro* drug release study with various kinetic models like Zero-order (cumulative percent drug release vs. time), First-order (log cumulative percent drug release vs. time), Higuchi’s kinetics (cumulative percent drug release vs. √time), and the Korsmeyer and Peppas equation (log cumulative percentage of drug release vs. log time) [22].

#### Stability Studies

A study was also carried out to assess the stability of sumatriptan succinate-loaded chitosan nanoparticles (CNP4). For this purpose, samples were kept in borosilicate glass vials and stored at room temperature, in a refrigerator (5 ± 1°C), and 45 ± 1°C (75% relative humidity) in the stability chamber. Samples were analyzed at the intervals of 0, 7, 14, 21, 28, 35, and 45 days for their drug content as well as any changes in physical appearance [23].

## Results

### Particle size, Surface Morphology, and Zeta Potential Measurement of Nanoparticles

The particle size and zeta potential of the chitosan nanoparticles were analyzed by PCS and a zetasizer. The nanoparticles were round in shape with a smooth appearance as shown in Figure 1. The values for the average particle size, zeta potential, and polydispersity index are tabulated in Table 3 and Figure 2. On the basis of Taguchi design, the “smaller-the-better” of S/N ratio was considered for the particle size and polydispersity index. CNP4 showed the minimum average particle size of 306.8 nm and a polydispersity index of 0.32, respectively, which can be taken into account as optimized in terms of particle size and polydispersity index. The zeta potential of the optimized formulation was found to be +28.5 mV.

### Entrapment Efficiency

Entrapment efficiency of the sumatriptan succinate-loaded chitosan nanoparticles was analyzed and the data are shown in Table 3. According to Taguchi design, the “larger-the-better” S/N ratio is considered optimum. CNP4 showed an average drug entrapment efficiency of 75.4 ± 1.1%, which is higher amongst the other formulations.

### In vitro Drug Release Study

*In vitro* drug release was carried out using the K-C (diffusion) cell. Figure 3 shows the release profile of sumatriptan succinate from chitosan nanoparticles. The cumulative release of the drug from the drug-loaded formulations varied between 55.1 ± 1.1% and 76.7 ± 1.3% for 28 hours, depending on the drug-polymer ratio.

### Release Kinetics

It was found that the *in vitro* drug release of CNP4 was best explained by Zero-order, as the plots showed the highest linearity (R^2^ = 0.986), followed by Higuchi’s equation (R^2^ = 0.959), and First-order (R^2^ = 0.723). The corresponding plot (log % cumulative drug release vs. log time) for the Korsmeyer-Peppas equation indicated good linearity (R^2^ = 0.924). The release exponent ‘n’ was found to be 0.631.

### Stability Studies

The results for the drug content of the optimized formulation CNP4 after 45 days of stability testing at different storage conditions are shown in Table 4.

## Discussion

According to the literature, chitosan nanoparticles can be prepared by several techniques such as the microemulsion method, the ionotropic gelation method, and the solvent emulsification diffusion method. Ionotropic gelation requires a simple laboratory set-up and was used in the present study by varying the amount of polymer (chitosan), cross-linking agent (sodium tripolyphosphate), and surfactant (Tween 80). Additionally, the pH was considered as an important factor as it determines the degree of cross-linking and also the pH of the delivery site. It was reported that cross-linking of the polymer is higher at an acidic pH as at pH 3 only phosphoric ions are present, which cause ionic cross-linking, while at pH 9 cross-linking via deprotonation is achieved [24]. Ionotropic gelation yielded remarkably high drug entrapment (75.4 ± 1.1%) and small particle size (306 nm).

The Taguchi design enabled the selection of an optimized formulation. Four factors (drug-polymer ratio, concentration of surfactant, concentration of cross-linking agent, and stirring speed) were selected as independent variables, since they govern the dependent variables of particle size, entrapment efficiency, and *in vitro* drug release. The range of the drug-polymer ratio was set from 1:0.1 to 1:1.5 (w/w). In comparison with other ratios, 1:0.1 yielded the highest entrapment (41.4 ± 0.6%) with the minimum particle size (415 ± 0.2 nm). Out of the different concentrations of the cross-linking agent in the range from 0.10 to 0.75%, 0.25% was found to be the best on the basis of smallest particle size (506 ± 0.7 nm) and high entrapment (56.2 ± 0.4%). The concentration of surfactant i.e. 0.5% (v/v) Tween 80 was selected on the basis of the smallest particle size (415 ± 0.2 nm) as well as the fact that no aggregation was observed for up to 24 hours. A stirring speed of 1500 rpm was selected as the optimum with respect to particle size (415 ± 0.4 nm) and entrapment efficiency (52.8 ± 0.4%).

On the basis of the evaluated parameters, the optimized formulation should have a medium level of drug-polymer ratio (1:0.2, w/w), low level of surfactant (0.25%, v/v), medium level of cross-linking agent (0.25% w/v), and a high level of stirring speed (3000rpm), as concluded from the three levels of factors selected for experimental design.

The shape and surface morphology of the sumatriptan-loaded chitosan nanoparticles were visualized by scanning electron microscopy (SEM) and revealed a spherical shape and with a smooth surface (Figure 1). Furthermore, chitosan nanoparticles were nearly uniform in their size distribution. Their average particle diameter was 306.8 nm with a polydispersity index of 0.3. This may be attributed to the optimum selection of drug-polymer ratio and stirring speed. The polymer solution (0.2%-medium level) was not too viscous, thus a high stirring speed (3000rpm) could easily break down the formed droplets. As reported in the literature, a higher concentration of polymer results in more viscous solutions which may resist particle breakdown by stirring and lead to an increase in particle size [25]. A smaller size helps in targeting and increasing the drug’s penetration of biological membranes [26]. The measurement of the zeta potential allows predictions of storage stability of colloidal dispersions. In general, particle aggregation is less likely to occur in cases of high zeta potential due to electric repulsion [3]. The mean zeta potential was found to be +28.79 mV which may be attributed to the positive charges on the polymer’s matrices and surfactant’s mixture. Additionally, Tween 80 also provides steric stabilization of the nanoparticles [27].

The drug entrapment was relatively high in the formulation CNP4 amounting to 75.5 ± 1.1%, as compared to the other formulations. Effective entrapment depends on the type of polymer and solubility of the drug in the polymer. Since chitosan is a hydrophilic polymer and sumatriptan succinate is also freely soluble in water, more of the drug could be entrapped into the polymer matrices. Apart from the hydrophilicity of the drug and polymer, the concentration of polymer (medium level) was high enough to entrap high amounts of drug, which led to increased entrapment [25]. If the concentration of the polymer is further increased, a decrease in entrapment is observed, which may be due to a higher viscosity of the polymeric solution which hinders diffusion of the drug into the polymer. Despite the optimum concentration of chitosan, the concentration of the cross-linking agent played a major role in entrapment efficiency. Higher concentrations of the cross-linking agent could gelate a higher amount of polymer, thereby increasing the amount of drug entrapped into the nanoparticles [28].

The *in vitro* release profile of the drug-loaded chitosan nanoparticles (Figure 3) revealed that the optimized batch showed an initial burst release up to 4.4 ± 1.9%, which may be due to the presence of adsorbed drug on the surface of the polymer, which was followed by sustained release of 76.7 ± 1.3% within 28 hours reflecting slow diffusion of the drug from the core to the surface. This release pattern may contribute to the presence of the drug immediately after instilling it in the nasal cavity and its sustained release up to 24 hours, which would contribute to lower dosing frequency. The slow release of the sumatriptan succinate from all formulations suggests homogeneous entrapment of the drug throughout the systems.

Upon fitting the *in vitro* release data into different equations, the optimized formulation showed Zero-order release as it has high linearity, followed by Higuchi’s equation and First-order as shown in Table 5. The value of release component ‘n’ obtained using the Korsmeyer-Peppas equation is 0.631 which appears to indicate the anomalous, non-Fickian diffusion suggesting that the drug release is controlled by more than one process i.e. superposition of both phenomena, the diffusion-controlled and swelling-controlled release [29].

The results of the stability studies clearly demonstrate that the formulation is more stable when stored at 5°C and room temperature than at 45 ± 2°C/75% RH. This may be due to nanoparticle degradation at higher RH and temperature [29].

## Conclusion

Sumatriptan succinate-loaded chitosan nanoparticles were successfully formulated via the ionotropic gelation technique using the Taguchi design for optimization. The obtained nanoparticles easily penetrate the nasal mucosa by virtue of particle size. The formulation displayed sustained release up to 24 hours which may help to reduce multiple daily doses to once per day.

## Figures and Tables

**Tab. 1:** Experimental control factors and their levels for chitosan nanoparticles formulation

S.No.	Variables	Levels		
		
		Low (1)	Medium (2)	High(3)
1.	Drug: Polymer Concentration	1:0.1%	1:0.2%	1:1.5%
2.	Concentration of Tween 80 (v/v)	0.25%	0.50%	0.75%
3.	Concentration of TPP (w/v)	0.1%	0.25%	0.5%
4.	Stirring speed (rpm)	1000	1500	2000

**Tab. 2:** Taguchi L_9_ (3^4^) orthogonal array for chitosan nanoparticles formulation

Batches	Parameters			
	
	A	B	C	D
CNP1	1	1	1	1
CNP2	1	2	2	2
CNP3	1	3	3	3
CNP4	2	1	2	3
CNP5	2	2	3	1
CNP6	2	3	1	2
CNP7	3	1	3	2
CNP8	3	2	1	3
CNP9	3	3	2	1

**Tab. 3:** Data for Particle Size and Entrapment Efficiency

Formulation	Average particle size ± S.D	Zeta Potential ± S.D	Polydispersity Index ± S.D	Entrapment Efficiency ± S.D
CNP1	556.2 ± 3.6	52.5 ± 0.2	0.50 ± 0.94	71.0 ± 2.8
CNP2	515.32 ± 3.5	21.71 ± 0.6	0.53 ± 0.82	58.1 ± 2.2
CNP3	444.67 ± 3.9	22.98 ± 1.2	0.49 ± 1.23	27.9 ± 0.8
CNP4	306.8 ± 3.9	28.7 ± 2.3	0.32 ± 1.09	75.4 ± 1.1
CNP5	376.9 ± 9.8	27.5 ± 0.9	0.34 ± 0. 34	66.6 ± 0.8
CNP6	650.48 ± 4.6	27.1 ± 1.0	0.64 ± 0.74	60.2 ± 1.2
CNP7	684.55 ± 5.3	26.8 ± 0.7	0.78 ± 0.32	52.8 ± 1.3
CNP8	580.03 ± 2.9	36.6 ± 1.4	0.68 ± 1.98	34.6 ± 1.1
CNP9	527.4 ± 5.3	37.78 ± 0.6	0.51 ± 0.59	49.1 ± 0.4

n=3; Values are mean ± standard deviation.

**Tab. 4:** Stability Studies of Optimized Formulation CNP4

S. No.	Sampling Interval (days)	Drug Content (%)		

		5 ± 1°C	Room temperature	45 ± 2°C/75%RH
1.	0	100	100	100
2.	7	99.5 ± 0.2	99.4 ± 0.2	92.5 ± 0.4
3.	14	99.2 ± 0.1	98.8 ± 0.1	85.9 ± 0.3
4.	21	99.9 ± 0.8	98.2 ± 0.1	76.83 ± 0.4
5.	28	99.7 ± 0.1	97.9 ± 0.2	70.97 ± 0.3
6.	35	98.4 ± 0.1	97.0 ± 0.1	63.86 ± 0.1
7.	45	98.2 ± 0.0	96.6 ± 0.3	57.03 ± 0.8

n=3; Values are mean ± standard deviation.

**Tab. 5:** Release parameters of chitosan nanoparticles (CNP4)

Formulation	Zero order		First order		Higuchi		Korsmeyer-Peppas	
	
	K	R^2^	K	R^2^	K	R^2^	N	R^2^
CNP1	2.671	0.971	0.057	0.698	13.55	0.969	0.757	0.727
CNP2	2.525	0.909	0.050	0.595	13.57	0.981	0.615	0.654
CNP3	2.588	0.985	0.057	0.723	12.96	0.958	0.706	0.670
CNP4	2.819	0.986	0.723	0.048	15.04	0.959	0.631	0.924
CNP5	2.616	0.987	0.058	0.683	14.25	0.966	0.972	0.865
CNP6	2.148	0.856	0.040	0.545	12.62	0.974	0.656	0.649
CNP7	2.075	0.948	0.054	0.654	11.67	0.988	0.986	0.844
CNP8	1.968	0.899	0.255	0.754	11.39	0.992	0.654	0.691
CNP9	2.238	0.966	0.051	0.631	12.31	0.964	0.865	0.825
