# Erratum to “Intranasal Delivery of Chitosan Nanoparticles for Migraine Therapy” [Sci Pharm. 2013; 81: 843–854]

**DOI:** 10.3797/scipharm.1208-18err

**Published:** 2013-10-06

**Authors:** Neha Gulati, Upendra Nagaich, Shubhini A. Saraf

**Affiliations:** 1 Department of Pharmaceutics, School of Pharmacy, Bharat Institute of Technology, Meerut, UP, India.; 2 School of Biosciences and Biotechnology, Babasaheb Bhimrao Ambedkar University, Lucknow, India.

**Keywords:** Erratum, Sci Pharm. 2013, 81: 843–854

## Abstract

This is an erratum to the article ‘Intranasal Delivery of Chitosan Nanoparticles for Migraine Therapy’ [Sci Pharm. 2013; 81: 843–854]. Figures 1–3 are added.

Unfortunately, all three figures were missing in the published article [1]. The authors and the editorial office are very sorry for this error and for any inconvenience this caused.

## Figures and Tables

**Fig. 1:**
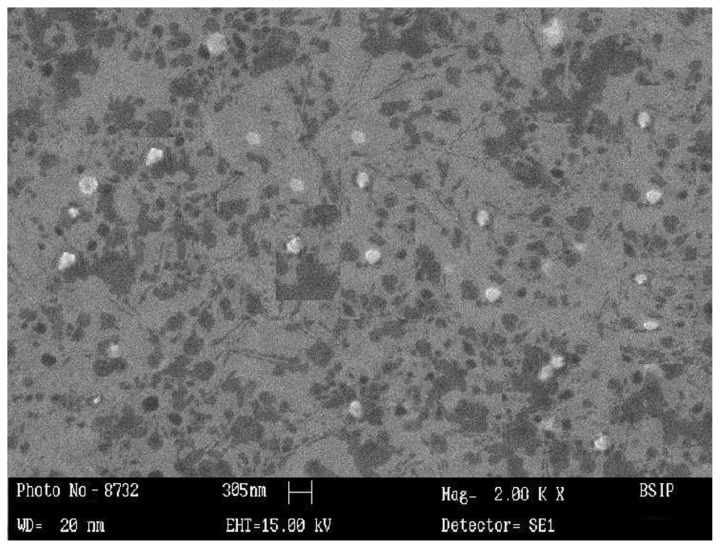
SEM Photomicrograph of sumatriptan succinate loaded Chitosan nanoparticles

**Fig. 2:**
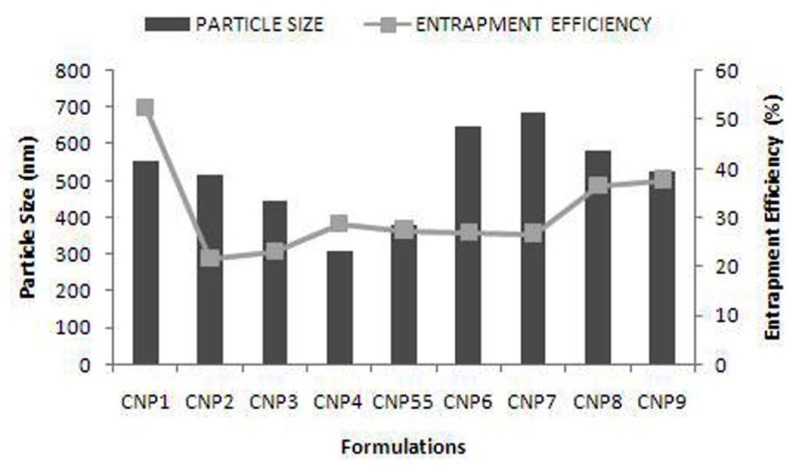
Particle size and Entrapment Efficiency of nine formulations

**Fig. 3:**
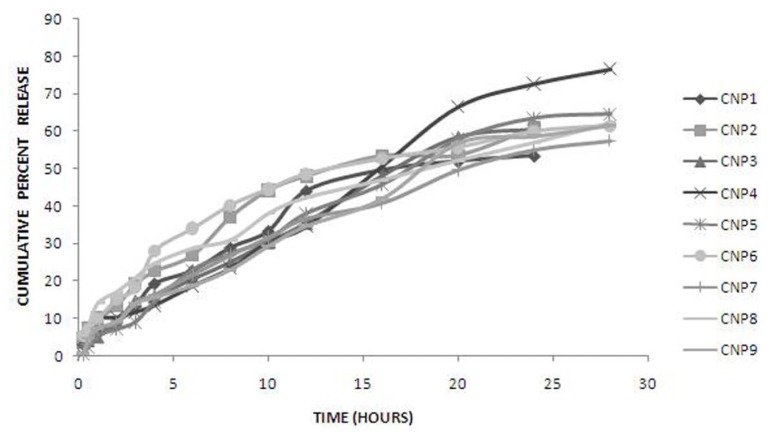
*In vitro* drug release profiles of sumatriptan loaded chitosan nanoparticles
